# Proteomic and Metabolomic Analysis of the Neuroprotective Effects of *Lycium Ruthenicum* Polyphenols in Diabetic Peripheral Neuropathy Mice

**DOI:** 10.1002/fsn3.70209

**Published:** 2025-05-01

**Authors:** Qi Tian, Hongdou Cao, Liwen Chu, Hua Gao, Qinghan Gao

**Affiliations:** ^1^ School of Public Health Ningxia Medical University Yinchuan Ningxia China; ^2^ Key Laboratory of Environmental Factors and Chronic Disease Control Ningxia Medical University Yinchuan Ningxia China; ^3^ Department of Pharmacy General Hospital of Ningxia Medical University Yinchuan Ningxia China

**Keywords:** diabetic peripheral neuropathy, *Lycium ruthenicum* polyphenols, Metabolomic, proteomic, sciatic nerve

## Abstract

*Lycium ruthenicum* polyphenols (LRP) have been proven to be anti‐inflammatory, antioxidant, and neuroprotective phytochemicals. This study applies proteomics and metabolomics to LRP‐treated db/db mice to explore its potential effects mechanism. The experiments were divided into three groups: normal control db/m group, diabetic peripheral neuropathy (DPN) db/db group, and LRP‐treated db/db group. We examined physiological and biochemical indicators, behavioral indicators, and histopathology. As for the mechanism, we used TMT‐based quantification proteomics and LC–MS/MS‐based metabolomics for sciatic nerve and serum. After 8 weeks of treatment, the fasting blood glucose level, mechanical withdrawal threshold, and thermal hyperalgesia were significantly improved. Pathological examination showed a significant alleviation in sciatic nerve histomorphology in the LRP group. Proteomics and metabolomics showed that the interventional effects of LRP were enriched mainly in oxidative phosphorylation, cardiac muscle contraction, and serum metabolites were enriched mainly in amino acid metabolism. LRP improves neurological function by improving mitochondrial functions, promoting neuronal development, and ameliorating dysregulation of amino acid metabolism. These results provide theoretical evidence for LRP as a potential functional food ingredient for the prevention and treatment of DPN.

## Graphical abstract

After 8 weeks of treatment, LRP showed multiple neuroprotective effects on nerve damage in the DPN model mice. The proteomics and metabolomics identified several possible mechanisms for their therapeutic effects: (1) improving mitochondrial function, especially improving the OXPHOS process; (2) increasing the abundance of cytoskeletal and structural proteins, promoting neuronal development, and synaptic plasticity to play the role of repair after nerve damage; (3) improving amino acid metabolism disorders, improving insulin resistance, and playing a role in lowering glucose.

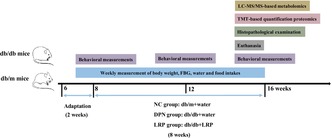

## 1 Introduction

Diabetic peripheral neuropathy (DPN) is a common complication of diabetes mellitus (DM) and it affects approximately 50%–60% of DM patients (Cernea and Raz 2021; Kaur et al. 2023). DPN is characterized by progressive degeneration of peripheral nerves from distal to proximal, comprising axonal dying‐back degeneration, fiber loss, segmental demyelination, myelin formation, and abnormal regenerative sprouting (Eid et al. 2023; Elafros et al. 2022; Yang et al. 2022). Clinical symptoms mainly are numbness, pain sensitivity, and sensory abnormalities, which are the main causes of disability and amputation in DM patients and seriously affect the quality of life (Pathak et al. 2022; Selvarajah et al. 2019).

However, the pathogenesis of DPN is still unclear. It is currently thought to be mainly related to hyperglycaemic pathways, lipid metabolism disorders, oxidative stress, nutritional deficiencies, microangiopathy, and abnormal insulin signaling pathways (Feldman et al. 2019; Sloan et al. 2021). Current clinical treatment of DPN is mainly focused on the control of blood glucose and lipids, and there are no specifically effective drugs available (Pop‐Busui et al. 2017). Therefore, developing new treatment strategies to slow the progression of DPN is necessary.

*Lycium ruthenicum* is a unique medicinal plant in western China, and its polyphenols are one of the main active ingredients (Wang et al. 2018). Polyphenols can be up to (20.17 ± 2.82) mg/g (Zhang et al. 2018). The study found that the polyphenols in *Lycium ruthenicum* mainly include two flavonoids, catechin and naringenin, and seven phenolic acids (Gamage et al. 2021; Liu et al. 2020). Various in vitro and in vivo studies have shown that polyphenols ameliorate hyperglycemia‐induced oxidative stress (Taïlé et al. 2020), inflammation (Yehuda et al. 2015), and apoptosis (An et al. 2020), reduce inflammatory markers (Millán et al. 2019; Mittal et al. 2018), improve peripheral nerve function indicators (Ding et al. 2014) and mitochondrial function (Naoi et al. 2019). Polyphenols may be a good dietary source for preventing nervous system disease. However, the protective molecular mechanisms of polyphenols are not fully understood. This study applied multi‐omics techniques to further elucidate the neuroprotective mechanisms of polyphenols.

Omics studies have rapidly developed in recent years, which can explain molecular changes at different levels, providing a more comprehensive understanding of the underlying mechanisms of drugs and diseases. Proteomics takes all the proteins encoded by the genome or tissue cells as the research object and conducts overall research on the pathogenesis, cell model, functional connection, etc. at the protein level (Altelaar et al. 2013; Li et al. 2017). Metabolomics studies the metabolic spectrum of endogenous small molecular weight metabolites in biological systems, which can not only comprehensively evaluate the chemical processes involving metabolites in systems biology, but also provide new insights into the overall efficacy (Astarita et al. 2023). This study uses proteomics and metabolomics analysis technology to identify the differential metabolites in the sciatic nerve protein and serum of diabetic peripheral neuropathy db/db control mice and LRP‐treated db/db mice to reveal the complex biological process and neuroprotective mechanism of LRP.

## 2 Materials and Methods

### 2.1 Materials and Chemicals

*Lycium ruthenicum* polyphenols (LRP) were purchased from Xi'an Qing Zhi Biotechnology Co., LTD. (Xi'an, China). Our previous studies have described the characterization and quantification of LRP using high‐performance liquid chromatography (HPLC), with the predominant phenolic compounds in LRP being rutin, p‐coumaric acid, catechin, and caffeic acid (Gao et al.^2020^). The extracts used in this study comply with national standards.

### 2.2 Animals and Research Design

Male BKS‐Lepr^*db/db*^ (*n* = 30, 6 weeks) and Lean littermate BKS‐Lepr^*db/m*^ (*n* = 15, 6 weeks) mice were obtained from Changzhou Cavens Laboratory Animal Technology (Quality Certificate NO. 202257009, Experimental Animals License SCXK (Su) 2021–0013). All mice were housed under a controlled SPF‐grade environment (temperature: 22°C ± 2°C; humidity: 55% ± 10%; with a 12/12 h light/dark cycle) with dietary drinking water available ad libitum. All animal procedures complied with the Guide for the Care and Use of Laboratory Animals, and the Ningxia Medical University Ethics Committee approved this study in June 2022 (ethical number: IACUC‐NYLAC‐2022‐187).

After 2 weeks of adaptive feeding with a standard laboratory diet, the db/db mice were randomly divided into the DPN group (diabetic peripheral neuropathy model group) and the LRP group (LRP intervention group) containing 15 mice each, and the db/m mice were divided into the NC group (normal control group) containing 15 mice. The intervention dose of LRP (100 mg/kg/day, 0.1 mL/10 g body weight) was selected based on preliminary studies from our research group (Pang et al. 2023) and administered daily via oral gavage to the mice for 8 consecutive weeks. The NC and DPN groups were administered water in the same way. After 8 weeks of treatment, the mice were sacrificed under anesthesia, and serum and sciatic nerve were collected for further analysis. Experiments were randomly grouped using Excel software to generate random numbers.

### 2.3 Behavioral Measurements

Mechanical withdrawal threshold was assessed using von Frey monofilaments, and tail flick time was recorded as measures of thermal latency (Shi et al. 2013; Zhang et al. 2020).

#### 2.3.1 Mechanical Withdrawal Threshold Testing

The mice were placed in a metal mesh with a 2 × 2 mm gap, and a Plexiglas box of fixed dimensions was used to separate each mouse. Prior to experimentation, the animals underwent a 30‐min acclimatization period in their novel environment, with limb movements gradually subsiding to infrequent levels. A series of calibrated von Frey filaments (0.4, 0.6, 1.0, 1.4, 2.0, 4.0, 6.0, 8.0 g) were applied vertically to the plantar surface of the hind paw, starting from the 0.4 g filament. Each filament had to be bent into a ‘C’ or ‘S’ shape and maintained for 6–8 s. The response of the mice to foot retraction was observed and recorded. The tactile response threshold was calculated as the average of the weights of the last and the previous filament (if the previous filament did not produce any response, the weight of the last filament was recorded). This was done separately for the right and left paws, and the average of the tactile response thresholds of the left and right paws was used for the final statistical analysis. The interval between pre‐ and post‐ stimulation was at least 10 min, and the interval between experiments was more than 4 days (DanMic Global.USA).

#### 2.3.2 Thermal Latency Testing

The experiments were carried out using a thermostatic water bath to adjust the temperature and stabilize it at 52°C ± 1.5°C (Tianjin Taisite Instrument Co. Ltd. China). The experimental operations were also conducted under the consistent temperature and humidity of the rearing environment. Firstly, the mice were placed in the immobilizer with their tails exposed. The tail‐flick test was then carried out after the tails were in a relatively relaxed state. To do this, the last 2 cm of the tail was rapidly immersed in hot water. The endpoint was defined as the tail rolling up and being completely withdrawn from the water, and the time of the tail flick was recorded. The water on the tail was then wiped dry, and the tail‐flick test was repeated three consecutive times. The time interval between each test was greater than 30 s, and the average of the three results was taken as the final thermal latency (TL) result.

### 2.4 Physiological and Biochemical Parameters

Two independent researchers observed and recorded daily body weight, mental status, coat color, and daily activities. Their 24‐h food and water intake were monitored and recorded weekly. Fasting blood glucose (FBG) was measured weekly at a fixed time, after 8 h of fasting, using a standard blood glucose meter with blood taken from the tail vein (Sinocare Inc. China).

### 2.5 Histopathological Examination

After 8 weeks of intervention, the mice were subjected to deep anesthesia with isoflurane (RWD Life Science, China). The left sciatic nerve was isolated and taken, and immediately placed in 4% paraformaldehyde for overnight fixation at 4°C. After fixation, the sciatic nerve specimen was cut into two segments, and longitudinal and transverse embedding sections were performed. After hematoxylin–eosin (HE) staining, the pathological structural changes of the sciatic nerve were observed under a light microscope (40×) (NikonEclipseE100, Japan).

Take the right sciatic nerve for isolation and extraction. Immediately after extraction, place it in a cold 2.5% glutaraldehyde electron microscope fixing solution, fix it overnight at 4°C, embed the section, and observe the ultrastructural changes and myelin sheath layer structure of the sciatic nerve under the electron microscope (5000× and 40,000×) (Nikon HITACHI HT7800, Japan).

### 2.6 Proteomic Analysis and Data Acquisition

Sciatic nerve tissue specimens were initially crushed and turned into a fine powder while submerged in liquid nitrogen. Subsequently, they were subjected to extraction using a urea lysis buffer, which contained 8 M urea and 1% SDS, along with the addition of a protease inhibitor. The samples were then lysed by keeping them on an ice bath for a duration of 30 min, during which they were intermittently mixed using a vortex mixer for 5–10 s at 5‐min intervals. Following this, the samples were centrifuged at a speed of 16,000 *g* at a temperature of 4°C for half an hour. The resulting supernatant was subsequently retrieved. The concentration of proteins within the samples was measured by employing a bicinchoninic acid (BCA) Protein Assay Kit. A quantity of 15 μg of total proteins from each sample was isolated and then fractionated by utilizing a 12% SDS‐PAGE gel. Subsequently, all the samples underwent trypsinization and were labeled for further analysis. High pH reverse‐phase chromatography was employed to divide the samples, aiming to enhance the extent of proteomic analysis. The peptide samples were re‐dissolved using an ultra‐performance liquid chromatography (UPLC) loading buffer, which consisted of 2% acetonitrile with ammonia adjusted to a pH of 10. Subsequently, the separation of the samples was carried out utilizing a reversed‐phase C18 column, specifically an ACQUITY UPLC BEH C18 Column with a particle size of 1.7 μm, and dimensions of 2.1 mm in width and 150 mm in length, as provided by Waters, USA. The two‐dimensional analytical procedure was executed through the use of liquid chromatography coupled with tandem mass spectrometry (LC–MS/MS), which was facilitated by the Evosep One system in conjunction with an Orbitrap Exploris 480 mass spectrometer.

The RAW data files were analyzed using Proteome Discoverer (Thermo Scientific, Version 2.4) against the Proteome Discoverer database. Precursor Mass Tolerance was set at 20 ppm and fragment Mass Tolerance was set at 0.02 Da. The false discovery rate (FDR) of peptide identification was set as FDR ≤ 0.01. A minimum of one unique peptide identification was used to support protein identification. The t‐test function in R was used to calculate the *p*‐value for the significance of the difference between samples, while the fold change (FC) was calculated between groups. The thresholds of fold change (FC > 1.2 or < 0.83) and *p*‐value < 0.05 were used to identify differentially expressed proteins (DEPs) (Li et al. 2022).

### 2.7 Metabolomic Analysis and Data Acquisition

A volume of 100 μL serum was pipetted into a 1.5 mL centrifuge tube and combined with 400 μL of a solvent mixture (acetonitrile to methanol in a 1:1 ratio by volume) containing an internal standard at a concentration of 0.02 mg/mL, L‐2‐chlorophenylalanine, for metabolite extraction. Subsequently, the mixture was vortexed for 30 s and subjected to low‐temperature sonication for 30 min at 5°C and a frequency of 40 KHz. After allowing the samples to precipitate proteins by cooling at −20°C for 30 min, centrifugation was performed at 4°C and 13,000 *g* for 15 min. The supernatant was carefully transferred to a fresh tube and evaporated to dryness under a stream of nitrogen. The dried residue was re‐dissolved in 100 μL of a reconstitution solution (acetonitrile to water in a 1:1 ratio), followed by a further extraction step involving low‐temperature sonication for 5 min at 5°C and 40 KHz, and then centrifuged again at 13,000 *g* and 4°C for 10 min. The supernatant was subsequently transferred to sample vials in preparation for liquid chromatography–tandem mass spectrometry (LC–MS/MS) analysis. As part of the system conditioning and quality control protocol, a pooled quality control (QC) sample was formulated by blending equal aliquots of all individual samples. These QC samples were processed and analyzed using the identical methodology applied to the experimental samples. The LC–MS/MS analysis was performed on a Thermo UHPLC‐Q Exactive HF‐X system, equipped with an ACQUITY HSS T3 column (100 × 2.1 mm i.d., 1.8 μm; Waters, USA) at Majorbio Bio‐Pharm Technology Co. Ltd. (Shanghai, China). The mobile phase system comprised two solvents: 0.1% formic acid in water: acetonitrile (95:5, v/v) designated as solvent A, and 0.1% formic acid in acetonitrile: isopropanol: water (47.5:47.5, v/v) designated as solvent B. For positive ion mode separation, the gradient elution was programmed as follows: from 0 to 3 min, a linear increase of mobile phase B from 0% to 20%; from 3 to 4.5 min, an increase from 20% to 35%; from 4.5 to 5 min, a further increase to 100%; held at 100% from 5 to 6.3 min; followed by a return to 0% from 6.3 to 6.4 min, and maintained at 0% from 6.4 to 8 min. For negative ion mode separation, the gradient profile was as follows: an initial increase of mobile phase B from 0% to 5% over 1.5 min; a rise to 10% by 2 min; an increase to 30% by 4.5 min; a linear ascent to 100% by 5 min; held at 100% from 5 to 6.3 min; a decrease to 0% from 6.3 to 6.4 min; and maintained at 0% from 6.4 to 8 min. The flow rate was set at 0.40 mL/min, with the column temperature regulated at 40°C. Mass spectrometric data acquisition was conducted utilizing a Thermo UHPLC‐Q Exactive HF‐X Mass Spectrometer coupled with an electrospray ionization (ESI) source, operating in both positive and negative modes. The data were collected using the Data Dependent Acquisition (DDA) mode, with a mass range spanning from 70 to 1050 m/z. The subsequent preprocessing of the raw LC/MS data was executed with the Progenesis QI software (Waters Corporation, Milford, USA), which facilitated the exportation of a comprehensive three‐dimensional data matrix in CSV format. This matrix encapsulated pertinent details, including sample descriptors, metabolite identities, and their respective mass spectral response intensities. Subsequently, the data matrix underwent rigorous curation, which involved the elimination of internal standard peaks and the exclusion of recognized false positives, such as spurious noise, column bleed, and peaks attributable to derivatization reagents. The data were then deduplicated and peak‐pooled to enhance analytical reliability. Parallel to these steps, metabolite identification was pursued through database interrogation, leveraging key databases including the HMDB, Metlin, and the Majorbio Database. These databases served as the primary resources for the annotation of metabolites, ensuring the accuracy and scientific validity of the findings (Wang et al. 2021).

The data matrix was searched with a database and uploaded to the Majorbio cloud platform for data analysis. Initially, the data matrix underwent preprocessing with the following outcomes: a retention of at least 80% of the metabolic features detected in each group of samples. Post‐filtering, for specific samples where metabolite levels were below the lower limit of quantification, the minimal metabolite value was estimated, and each metabolic feature was normalized to the sum. To mitigate errors arising from sample preparation and instrumental instability, the response intensities of the mass spectrometry peaks were normalized using the sum normalization method, yielding a normalized data matrix. Concurrently, variables from quality control (QC) samples with a relative standard deviation (RSD) exceeding 30% were excluded and subjected to log10 transformation, culminating in the final data matrix for subsequent analysis (Wang et al. 2021).

Subsequently, principal component analysis (PCA) and orthogonal partial least squares‐discriminant analysis (OPLS‐DA) were conducted utilizing the R software package “ropls” (Version 1.6.2), followed by a 7‐cycle interactive validation to assess the stability of the model. Based on the orthogonal partial least squares‐discriminant analysis (OPLS‐DA) model, metabolites were identified as significantly differential with a Variable Importance in the VIP ≥ 1, *p* < 0.05 as determined by the student's *t*‐test.

### 2.8 Bioinformatics Analysis

Principal component analysis (PCA) was used to ascertain the association of the groups of samples with each other. Volcano plots were drawn to visualize the expression of differential proteins and differential metabolites between different groups. Functional annotation and pathway analysis of DEPs and DEMs employed the GO database and the KEGG database.

### 2.9 Statistical Analyses

The outcomes were presented as the mean ± standard deviation (SD). Group comparisons were conducted using one‐way analysis of variance (ANOVA), supplemented by Tukey's test for multiple comparisons. A *p*‐value of less than 0.05 was deemed indicative of statistical significance. Statistical analyses were performed utilizing SPSS software (version 26.0).

## 3 Results

### 3.1 Biochemical Indicators

After 2 weeks of adaptive feeding, the average fasting blood glucose level (fasting 8–12 h) of db/db mice was approximately 11.1 mmol/L^−1^, which can be considered diabetic (Cui et al. 2023). They were then randomized into the LRP groups and DPN groups. Eventually, 15 mice in each of the NC, DPN, and LRP groups were included as study subjects.

**FIGURE 1:**
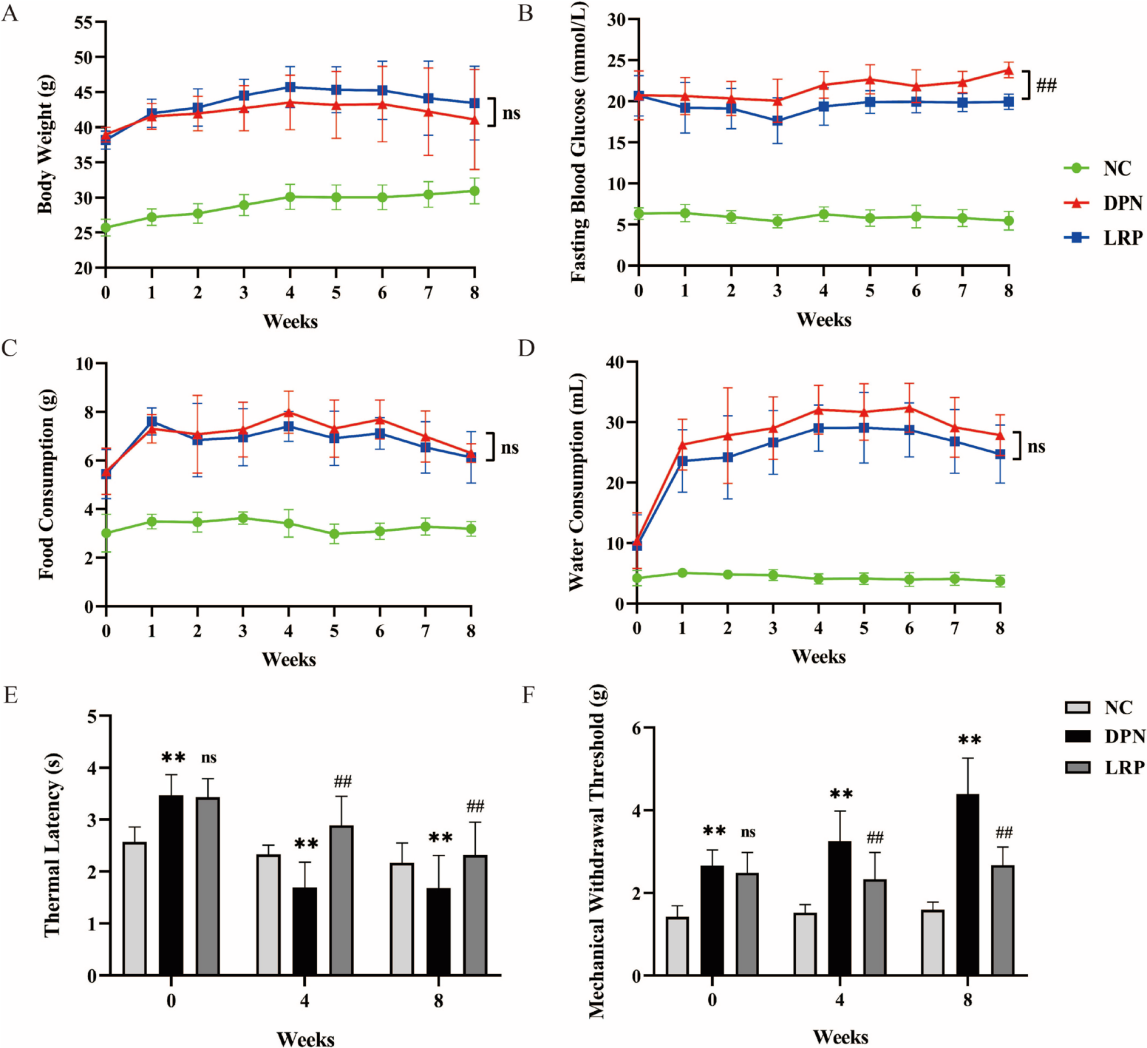
Effect of LRP on body weight (A), fasting blood glucose (FBG) (B), food consumption (C), water consumption (D), thermal latency (E), and mechanical withdrawal threshold (F). Data are group mean ± SD. **p < 0.01, NC group vs. DPN group; ^##^ *p* < 0.01, ^ns^ *P* > 0.05, DPN group vs. LRP group.

During the LRP intervention period, the body weight and blood glucose levels were recorded weekly. Compared with the control group mice, the levels of body weight, FBG, food intake, and water consumption of the db/db model group were increased significantly (Figure 1A–D). After LRP treatment, the mice in the LRP group showed a statistically significant decrease in fasting blood glucose (*p* < 0.01). Food consumption (*p* = 0.277), water consumption (*p* = 0.102), and body weight (*p* = 0.303) improved but were not statistically significant.

### 3.2 LRP Protected Against the Peripheral Neuropathy in DPN Mice

Diabetic Peripheral Neuropathy (DPN) is defined by the dysfunction of sensory and motor nerve dysfunction, especially affecting the sensory nerve at the distal end of the lower limbs. In db/db mice, the manifestations of mechanical allodynia and thermal hyperalgesia, such as increased latency of thermal withdrawal and elevated sciatic nerve mechanical withdrawal threshold, confirm the development of peripheral neuropathy. A significant elevation in thermal pain threshold was observed in the DPN group relative to the Normal Control (NC) group (Figure 1E, *p* < 0.01). Furthermore, intervention with LRP notably mitigated the abnormal mechanical hyperalgesia in comparison to the DPN group (Figure 1E, *p* < 0.01). Mechanical pain thresholds were significantly lower in the DPN model group than in healthy mice in the NC group (Figure 1F, *p* < 0.01), and significantly improved in the LRP intervention group compared with the DPN group (Figure 1F, *p* < 0.01). LRP administration markedly rescued the reduction of mechanical withdrawal threshold and thermal hyperalgesia.

### 3.3 LRP Improved the Neurological Morphology of Sciatic Nerves in DPN Mice

**FIGURE 2:**
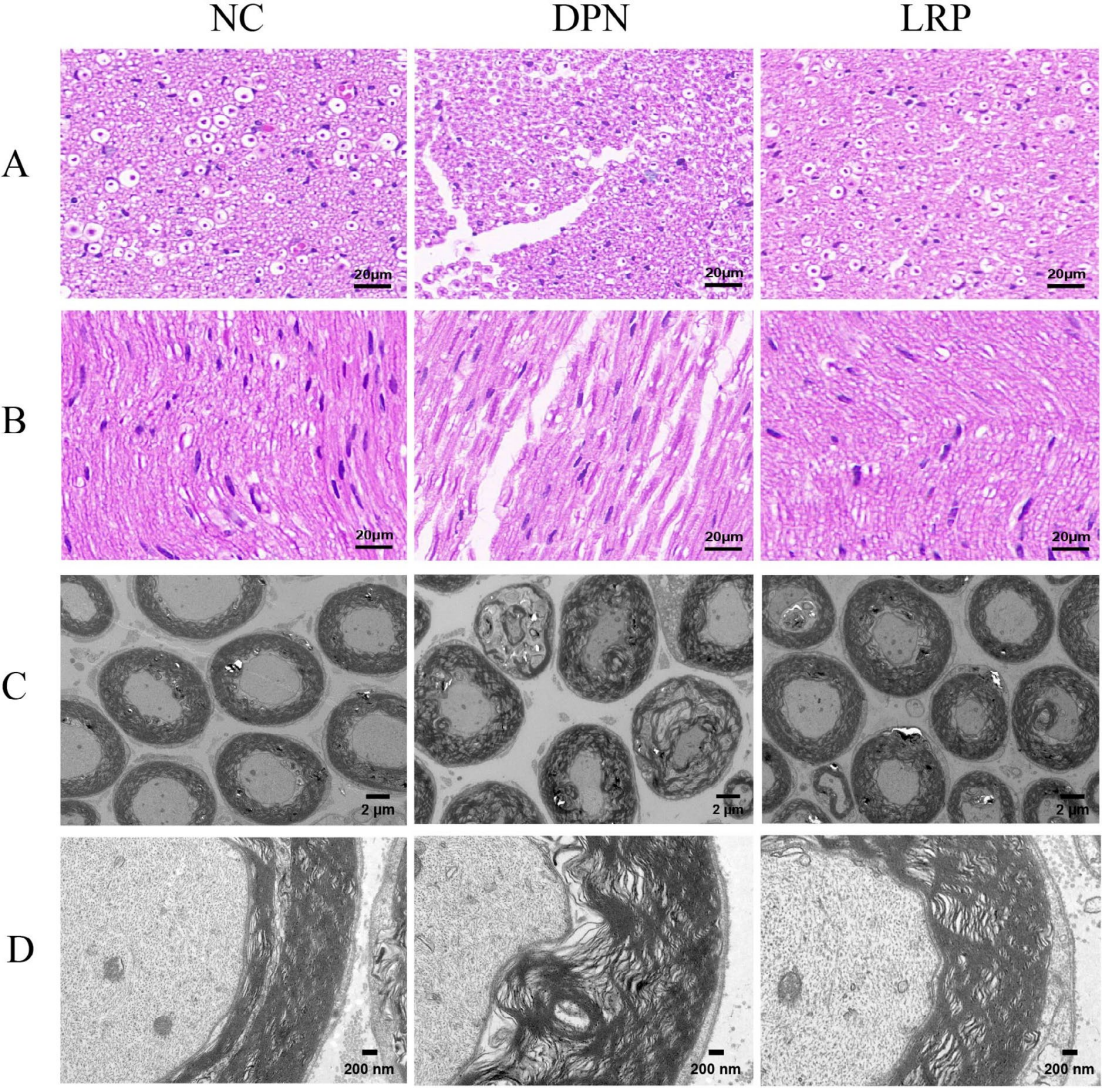
Histological examination of sciatic nerve in the cross‐sectional (A) and longitudinal (B) view Magnification: 40×, scale bars: 20 μm; Transmission electron microscopy sciatic nerves from different groups at magnifications of 5000× (C) and 40,000× (D) scale bars: 2 μm and 200 nm, respectively.

Hematoxylin and eosin (HE) staining depicted in Figure 2A,B, complemented by ultrastructural assessment via transmission electron microscopy (TEM) as shown in Figure 2C,D, revealed that the sciatic nerves of normal control mice featured myelinated fibers of diverse calibers with uniform distribution, regular profiles, and intact myelin sheaths of thickness commensurate with axonal diameters. In contrast, the DPN group mice exhibited myelinated fibers characterized by axonal atrophy and deformed myelin sheaths under light microscopy, sparsely distributed. Transmission electron microscopy revealed vacuole‐like changes in nerve fiber structure and plate separation, axonal atrophy and swelling, and deformation of myelin and axonal structures to form tumor‐like structures in DPN mice.

In LRP group mice, morphological aberrations were observed to be mitigated. The myelin lamina structure is well defined and uniformly wrapped around the nerve fibers. HE indicated that the myelin sheaths in LRP‐treated mice exhibited more regular morphologies and a more even distribution compared to those in DPN group mice. The pathological ultrastructural characteristics of the myelin and axons were ameliorated to varying extents by LRP treatment.

### 3.4 Proteomics Alternations

**FIGURE 3:**
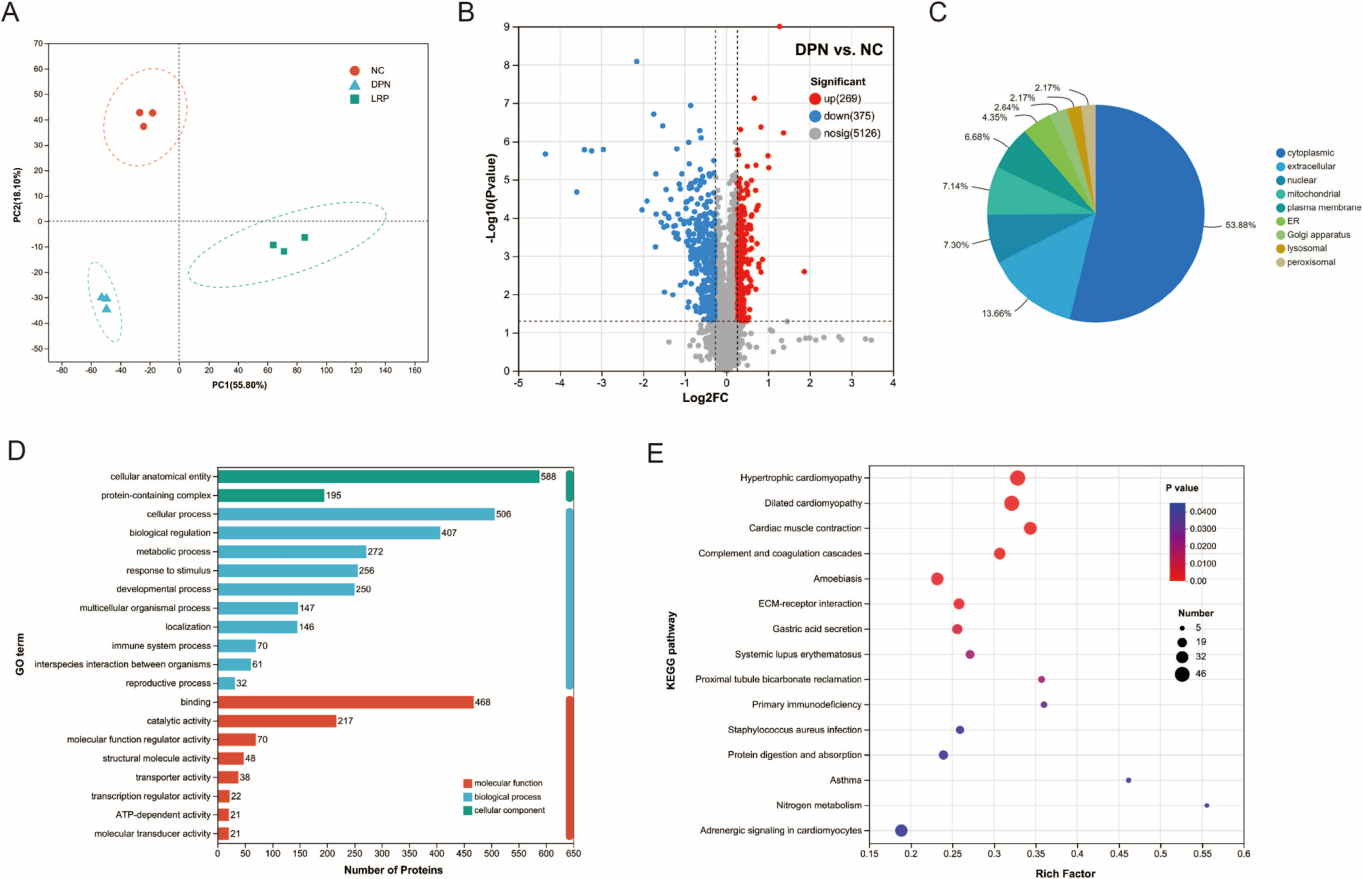
Proteome analysis of DPN and NC groups. (A) Principal component analysis (PCA) score plot; (B) Volcano plot of differentially expressed proteins; (C) Subcellular localization classification; (D) Gene Ontology (GO) classification;  (E) KEGG pathway enrichment analysis.

Based on the TMT‐based quantitative proteomics method, we detected 5770 proteins in the sciatic nerve samples (Figure 3A). Among them, 644 and 2159 DEPs were identified between the DPN vs. NC and LRP vs. DPN groups, respectively (FC > 1.2 or FC < 0.83; unpaired two‐sided student's *t*‐test, *p* < 0.05).

A total of 644 significantly different proteins were found in the DPN vs. NC group; 269 proteins were significantly upregulated and 375 proteins were significantly downregulated (Figure 3B). To determine the characteristics of the differentially abundant proteins, we annotated the Subcellular Localization, GO, and KEGG of the 644 proteins. Annotation of the Subcellular Localization showed that 53.88% of all identified significantly different proteins were localized to the cytoplasmic compartment, 13.66% to the extracellular space, 7.30% to the nuclear compartment, 7.14% to the mitochondrial compartment, 6.68% to the plasma membrane, 4.35% to the endoplasmic reticulum, 2.64% to the golgi apparatus, and 2.17% to the peroxisomal & lysosomal compartments (Figure 3C). GO functional annotation shows that most of the differentially abundant proteins participate in and regulate cellular anatomical entities and cellular processes through their binding (Figure 3D). GO enrichment analyses showed that BP classification indicated a variety of aberrant biological processes occurring in the DPN state, including muscle contraction, structural organization of myosin, regulation of lipid biosynthesis processes, organization of the actin cytoskeleton, inflammatory responses, modulation of interleukin‐10 production, negative regulation of lipid‐protein metabolism processes, and others. MF protein classification shows that most of the proteins bind to endopeptidase inhibitor activity, actin binding, calcium ion binding, cytoskeletal protein binding, insulin receptor activity, and carbohydrate binding (Supplementary Table S1). KEGG enrichment analysis showed that sciatic nerve difference proteins in DPN group mice were mainly involved in pathways including cardiac muscle contraction, nitrogen metabolism, protein digestion and uptake, steroid hormone biosynthesis, and adrenergic signaling in cardiomyocytes (Figure 3E).

**FIGURE 4:**
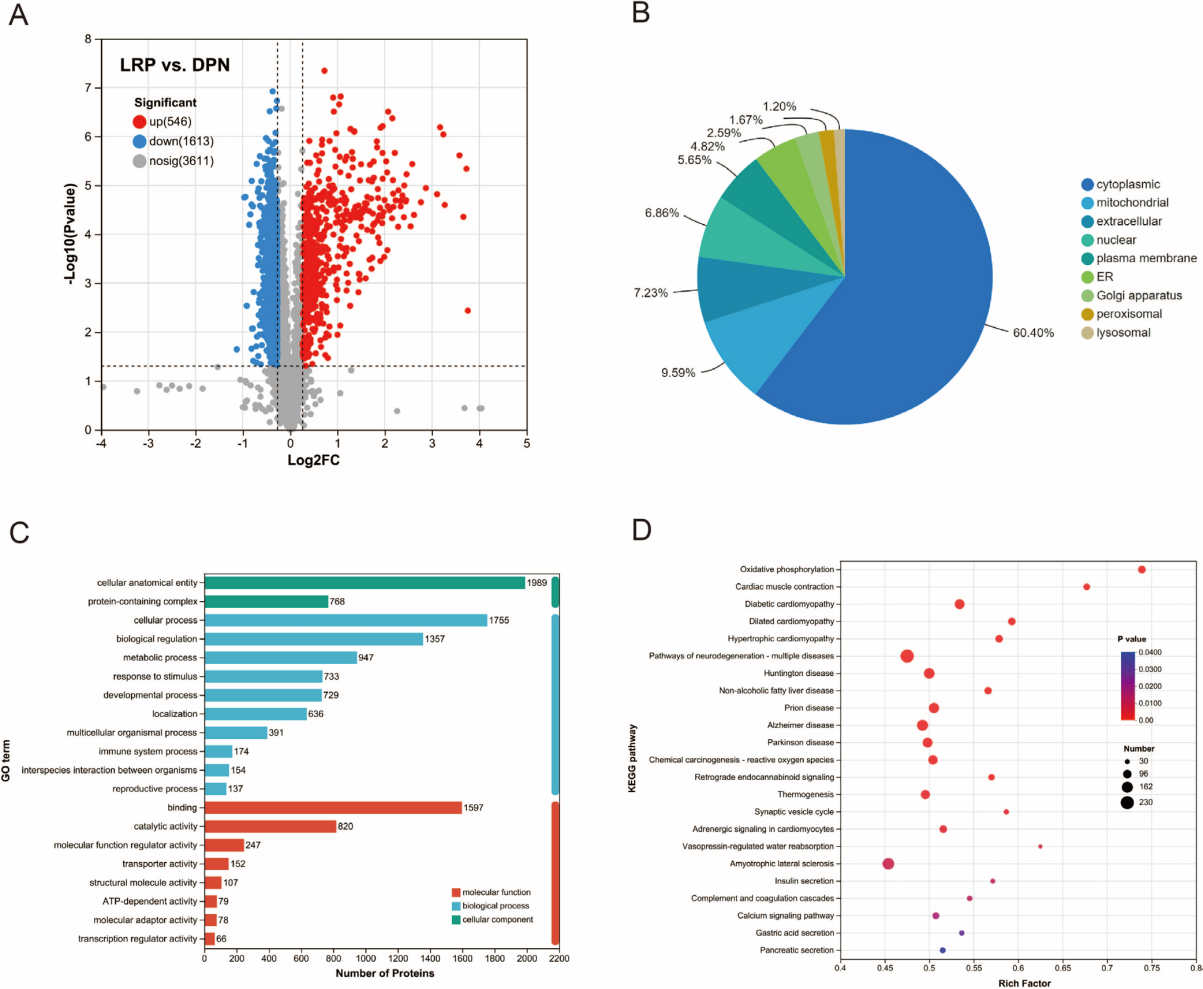
Proteome analysis of LRP and DPN groups. (A) Volcano plot of differentially expressed proteins; (B) Subcellular localization classification; (C) Gene Ontology (GO) classification; (D) KEGG pathway enrichment analysis.

A total of 2159 significantly different proteins were found in the LRP vs. DPN group; 546 proteins were significantly up‐regulated and 1613 proteins were significantly down‐regulated (Figure 4A). To determine the characteristics of the differentially abundant proteins, we annotated the Subcellular Localization, GO, and KEGG of the 2159 proteins. Annotation of the Subcellular Localization showed that 60.40% of all identified significantly different proteins were localized to the cytoplasmic, 9.59% to the mitochondrial, 7.23% to the extracellular, 6.86% to the nuclear, 5.65% to the plasma membrane, 4.82% to the endoplasmic reticulum, 2.59% to the Golgi apparatus, 1.67% to the peroxisomal, and 1.20% to the lysosomal (Figure 4B). GO functional annotation shows that most differentially abundant proteins participate in and regulate cellular anatomical entities and cellular processes through their binding (Figure 4C). GO enrichment analyses showed that BP classification indicated that LRP improvement of DPN mainly involved the purine ribonucleotide metabolic process, ribonucleoside triphosphate metabolic process, sarcomere organization, nucleoside triphosphate metabolic process, muscle system process, generation of precursor metabolites and energy, ribonucleotide metabolic process, regulation of exocytosis, ATP metabolic process, NADH dehydrogenase complex assembly, cytoskeleton organization, mitochondrial electron transport, NADH to ubiquinone, regulation of trans‐synaptic signaling, axon regeneration, and carbohydrate derivative biosynthetic process. MF protein classification shows most proteins with oxidoreduction‐driven active transmembrane transporter activity, actin binding, cytoskeletal protein binding, lipid binding, protein binding, calcium ion binding, serine‐type endopeptidase inhibitor activity, calcium‐dependent protein binding, GTPase activity, actin filament binding, signaling receptor binding, phospholipid binding, oxidoreductase activity, acting on NAD(P)H (Table S2). KEGG enrichment analysis showed that LRP improved differential proteins in DPN mice, mainly involved in pathways including oxidative phosphorylation, cardiac muscle contraction, diabetic cardiomyopathy, pathways of neurodegeneration‐multiple diseases, insulin secretion, calcium signaling pathway, and pancreatic secretion (Figure 4D).

### 3.5 Metabolomics Alterations

**FIGURE 5:**
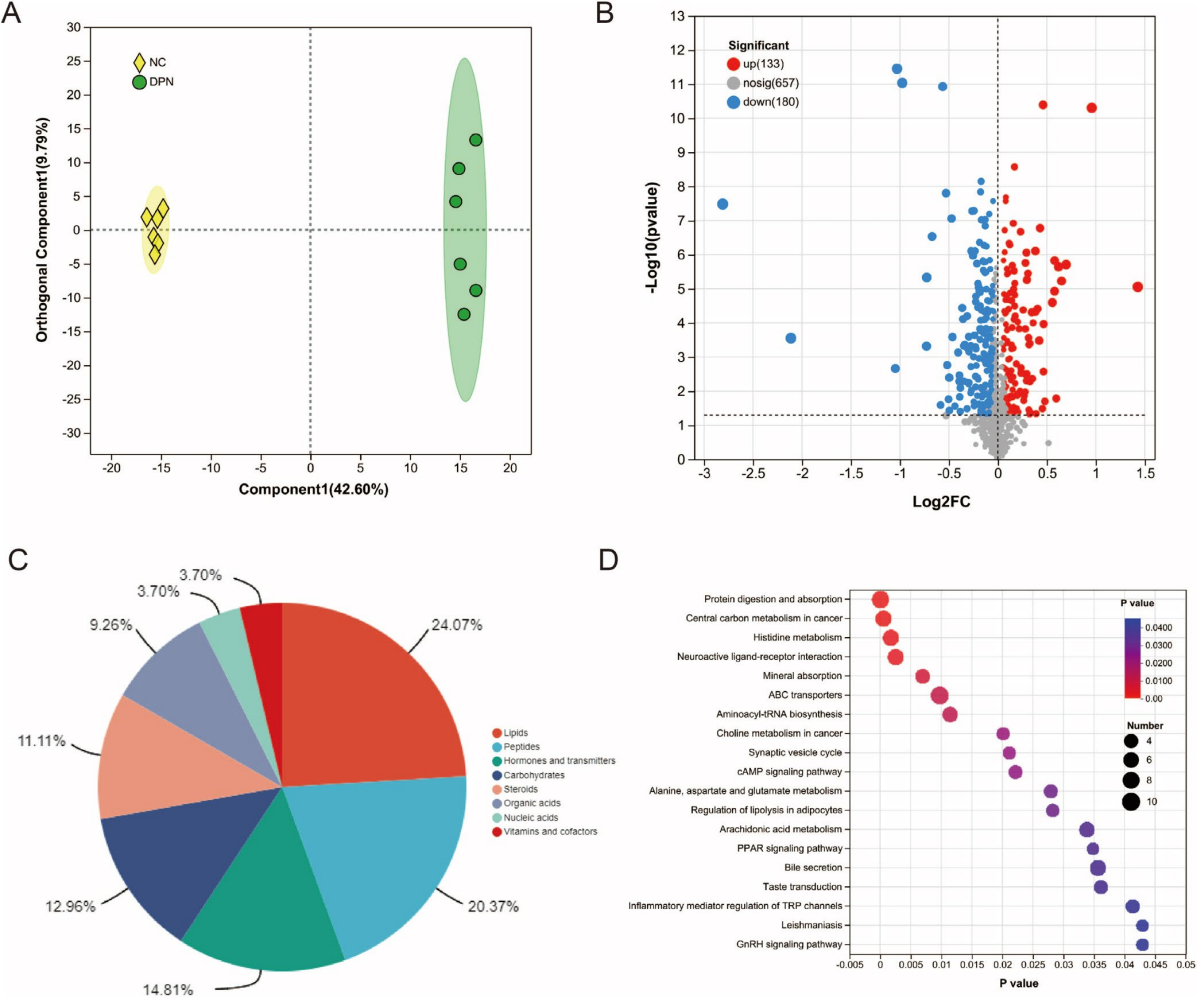
Metabolomics analysis of DPN and NC groups. (A) OPLS‐DA score plot; (B) Volcano plot of differentially expressed metabolites; (C) KEGG compound classification; (D) KEGG pathway enrichment analysis.

We compared the serum metabolomics between the DPN group and the NC group. The OPLS‐DA model shows a significant difference in the distribution of metabolites between the NC group and the DPN group (Figure 5A). According to the screening criteria of VIP ≥ 1, *p* < 0.05, 313 significant DEMs were identified between the DPN and NC groups in the serum; 133 metabolites were significantly up‐regulated and 180 metabolites were significantly down‐regulated (Figure 5B). KEGG compound classification categorizes 313 metabolites into 8 major categories, including lipids (24.07%), peptides (20.37%), hormones and transmitters (14.81%), carbohydrates (12.96%), steroids (11.11%), organic acids (9.26%), nucleic acids (3.70%), vitamins and cofactors (3.7%) (Figure 5C). KEGG pathway enrichment shows that differential metabolites in DPN mouse serum are mainly enriched in protein digestion and absorption, histidine metabolism, neuroactive ligand‐receptor interaction, mineral absorption, ABC transporters, aminoacyl‐tRNA biosynthesis, cAMP signaling pathway, alanine, aspartate and glutamate metabolism, regulation of lipolysis in adipocytes, arachidonic acid metabolism, PPAR signaling pathway, inflammatory mediator regulation of TRP channels, and GnRH signaling pathway (Figure 5D).

**FIGURE 6:**
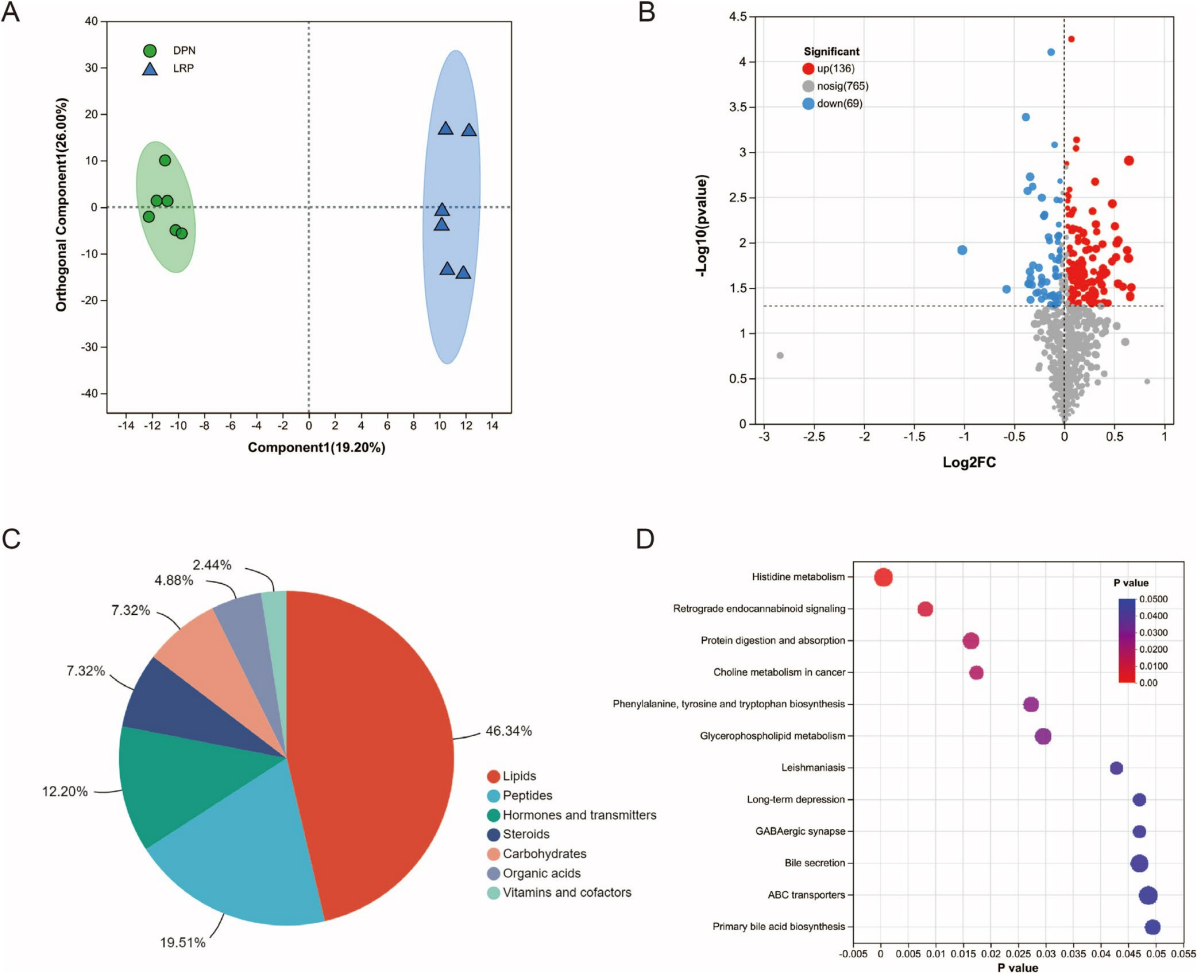
Metabolomics analysis of LRP and DPN groups. (A) OPLS‐DA score plot; (B) Volcano plot of differentially expressed metabolites; (C) KEGG compound classification; (D) KEGG pathway enrichment analysis.

A total of 205 serum metabolites were found in the LRP and DPN groups. The OPLS‐DA model shows a significant difference in the distribution of metabolites between the LRP group and the DPN group (Figure 6A). According to the screening criteria of VIP ≥ 1, *p* < 0.05, 205 significant DEMs were identified between the DPN and NC groups in the serum; 136 metabolites were significantly up‐regulated and 69 metabolites were significantly down‐regulated (Figure 6B). The KEGG compound classification categorizes 205 metabolites into 7 major categories, including lipids (46.34%), peptides (19.51%), hormones and transmitters (12.20%), steroids (7.32%), carbohydrates (7.32%), organic acids (4.88%), vitamins and cofactors (2.44%) (Figure 6C). The KEGG enrichment pathway shows that the intervention effect of LRP on DPN mice is mainly enriched in histidine metabolism, retrograde endocannabinoid signaling, protein digestion and absorption, choline metabolism in cancer, phenylalanine, tyrosine and tryptophan biosynthesis, glycerophospholipid metabolism, leishmaniasis, long‐term depression, GABAergic synapse, ABC transporters, and primary bile acid biosynthesis (Figure 6D).

## 4 Discussion

Diabetic peripheral neuropathy results in severe nerve damage, bringing huge pain and financial burden to patients; new preventive and therapeutic strategies are imperative. Polyphenols are commonly used worldwide as natural antioxidants and have been shown to have multiple beneficial effects. Db/db mice with severe obesity, hyperinsulinemia, and hyperglycemia are commonly used experimental models of type 2 diabetes (Hinder et al. 2013). The study used TMT‐based quantification proteomics and LC–MS/MS‐based metabolic analyses to explore the mechanistic effects of LRP on db/db mice.

This study administered LRP to db/db mice by gavage at a dose of 100 mg/kg/day for 8 weeks. Compared with db/m mice, the db/db mice displayed elevated body weight and blood glucose levels throughout the intervention. Body weight, food, and water intake showed no significant differences between the DPN and LRP groups, but the body weight of the LRP group was higher compared with the DPN group. A large number of studies have shown that diabetic models drink and eat heavily with significant weight loss (Gonçalves et al. 2022), and our study found that LRP improved abnormal physiological symptoms. Blood glucose was not significantly different in the first 2 weeks of the LRP intervention (*p* = 0.147), but was significantly lower in the LRP group than in the DPN group after 3 weeks of the intervention (*p* < 0.01). Mechanical withdrawal threshold and thermal hyperalgesia also had significant improvements after 4 weeks of intervention (*p* < 0.01). Furthermore, HE and electron microscopy showed that LRP improved sciatic nerve tissue (including myelin and axon morphology) to different degrees in DPN mice. LRP has shown beneficial effects on multiple parameters involved in diabetic neuropathy.

To further explore the potential mechanisms underlying their effects using proteomics and metabolomic analyses. Proteomic and metabolomic analyses revealed that LRP effectively altered sciatic nerve proteomic and serum metabolic profiles in db/db mice. Our study observed that LRP sciatic nerve proteins were enriched mainly in oxidative phosphorylation, cytoskeleton, and structure; LRP serum metabolites were enriched mainly in amino acid metabolism.

Proteomics functional enrichment analysis of sciatic nerve tissue showed that LRP intervention was deeply enriched with proteins related to oxidative phosphorylation, purine nucleotide metabolic process, and ribonucleotide metabolic process. The oxidative phosphorylation pathway was significantly increased in the LRP group compared to the DPN group. LRP significantly increased proteins involved in the mitochondrial respiratory chain, such as Cox7A1 and Cox6A2, suggesting that LRP regulates oxidative phosphorylation activity, supplies energy to cells, reduces ROS production, and prevents high glucose‐induced neuronal cell injury. Similarly, Zheng also showed that oxidative phosphorylation is impaired in the diabetic state and reactive oxygen species increase, increasing the progression of diabetes (Zheng and Qu 2022). Our results show that cytochrome c oxidase factors, including Cox7A1, Cox6A2, Cox1, Cox4, Cox5A, Cox5B, and Cox15, were significantly up‐regulated after LRP intervention, as were NADH–ubiquinone oxidoreductase (Complex I) factors, including Ndufaf7, Ndufv3, and ndufa13, which are all part of the mitochondrial respiratory chain and play important roles in energy transfer. Aberrant function of mitochondrial Cox and NADH oxidoreductases induces overproduction of ROS. Blocking NADPH oxidoreductase scavenges ROS to reduce oxidative phosphorylation and inhibit cell damage and even apoptosis. Therefore, LRP intervention regulates Cox and NADH oxidoreductase, which improves mitochondrial function to protect neuroprotective effects.

The study found other possible mechanisms for the effect of LRP. Sciatic nerve proteomics showed a significant increase in LRP group myosin, TNN13, MYL7, MYL3, TNNC, MYBPC2, ACTN2, and KEGG enrichment analyses showed that these proteins were mainly enriched in pathways such as cardiac contraction, diabetic cardiomyopathy, and dilated heart disease. These proteins are mainly involved in constituting muscle tissue and play a key role in muscle contraction. Myosin has ATPase activity and supplies muscle energy by cleaving ATP to release chemical energy. In addition, these proteins are involved in the formation of the myelin sheath, cytoskeleton, and axon, which play an important role in neuron development, axon transport, axon regeneration, and synaptic plasticity. Yang reported that diabetes resulted in axonal transport deficits and cytoskeletal alterations, exacerbating neuronal damage and worsening DPN onset and progression (Yang et al. 2023). LRP intervention increases the abundance of cytoskeletal and structural proteins, promotes neuronal and myelin regeneration, ameliorates nerve damage, and affects nerve injury protection.

Metabolomics functional enrichment analysis of serum showed that LRP intervention altered the metabolism of multiple amino acids in the LRP group, including four metabolites: histidine, arginine, taurine, and nicotinamide. Amino acids, as components of proteins, perform a variety of important physiological functions in organisms. Histidine‐related metabolites are up‐regulated after LRP treatment, and histidine can improve insulin resistance and inhibit inflammation and oxidative stress (Lent‐Schochet et al. 2019). L‐Arginine demonstrates multifaceted metabolic regulatory capacities, including maintaining glucose homeostasis, enhancing insulin sensitivity via nitric oxide (NO)‐mediated improvement of vasodilatory responses and glucokinase activity, preserving pancreatic β‐cell integrity, mitigating diabetes‐induced oxidative stress, and attenuating insulin resistance (Hu et al. 2017). Taurine has important antioxidant and apoptosis‐reducing properties (Gao et al. 2020). Nicotinamide can provide important neuronal and cytoprotective effects in diabetes and was found to exert neuroprotective effects by maintaining normal fasting blood glucose and reducing peripheral nerve damage induced by elevated glucose in diabetic animals (Kiss et al. 2020; Mandal et al. 2021). Zhou et al. demonstrated that dysregulation of amino acid metabolism not only contributes to the development of insulin resistance and the manifestation of type 2 diabetes (T2D) phenotypes but may also participate in the pathogenic mechanisms underlying diabetes mellitus progression (Zhou et al. 2022). Our results showed that LRP improves the disorder of amino acid metabolism in DPN mice. LRP upregulates multiple amino acid metabolites in DPN mice, which may protect diabetic peripheral neuropathy by maintaining glucose homeostasis in vivo, protecting neurons, and reducing oxidative stress. In the future, we will conduct further studies on key proteins and metabolites.

## 5 Conclusions

After 8 weeks of treatment, LRP showed multiple neuroprotective effects on sciatic nerve tissue in db/db mice. Proteomics and metabolomics identified several possible mechanisms for their therapeutic effects: (1) improving mitochondrial function, especially improving the OXPHOS process; (2) increasing the abundance of cytoskeletal and structural proteins, promoting neuronal development, and synaptic plasticity to play a role in repair after nerve damage; (3) improving amino acid metabolism disorders, improving insulin resistance, and playing a role in lowering glucose. This study reveals the potential clinical impact of using dietary LRP for the prevention and treatment of DPN.

## Author Contributions

**Qi Tian:** data curation (equal), methodology (equal), writing – original draft (equal). **Hongdou Cao:** software (equal), validation (equal). **Liwen Chu:** methodology (equal), visualization (equal). **Hua Gao:** conceptualization (equal), validation (equal). **Qinghan Gao:** supervision (equal), writing – review and editing (equal).

## Ethics Statement

All animal procedures complied with the Guide for the Care and Use of Laboratory Animals, and the Ningxia Medical University Ethics Committee approved this study in June 2022 (ethical number: IACUC‐NYLAC‐2022‐187).

## Conflicts of Interest

The authors declare no conflicts of interest.

## Supporting information


**Table S1.** GO enrichment analyses table of DPN and NC groups.

**Table S2.** GO enrichment analyses table of LRP and DPN groups.

## Data Availability

The data that support the findings of this study are available to the corresponding author upon reasonable request. The original number can be obtained at https://www.iprox.cn/page/PSV023.html;?url=1713363246367EKZe.
